# Midwives’ experiences with simulation-based education for postpartum hemorrhage management: a qualitative study

**DOI:** 10.1186/s41077-026-00415-0

**Published:** 2026-02-02

**Authors:** Ayşe Nur Ataş, Hilal Gizem Dalgıç, Yasemin Erkal Aksoy, Sema Dereli Yılmaz

**Affiliations:** Faculty of Health Science Midwifery Department Aladdin Keykubat Campus Selcuklu, Selcuk University, Konya, Türkiye

**Keywords:** Education, Qualitative, Midwife, Postpartum hemorrhage, Simulation

## Abstract

**Objectives:**

To determine the experience of midwives in a master's program undertaking simulation-based education for postpartum hemorrhage management.

**Methods:**

This is a qualitative study using a descriptive phenomenological approach. The study was conducted at Selçuk University, Selçuk Simulation Center between December 15–16, 2022. Purposive sampling was used to recruit 15 midwives who were students in a master's program. Simulation-based education for postpartum hemorrhage management was applied with scenarios. Data were collected using a semi-structured interview with midwives using an in-depth bilateral interview technique. All interviews were transcribed into an electronic text file.

**Results:**

According to the midwives' views on the simulation-based education experience, four main themes were developed: simulation experience, awareness formation, emotional effects, and the effects of the team members.

**Conclusions:**

Midwives described that simulation-based education in postpartum hemorrhage management increased their awareness and confidence, provided valuable experiential learning, elicited emotional responses such as stress and happiness, and fostered collaboration among team members.

## Introduction

Postpartum hemorrhage (PPH) is the worldwide leading cause of maternal mortality [1] and is reported to affect 10% of all births [2]. Early, timely, and precise identification of PPH is vital [2, 3]. Inaccuracies in assessment may lead to delayed recognition of PPH, delayed initiation of treatment, morbidity, and mortality [2]. PPH also leads to preventable maternal deaths, often due to delays in diagnosis, poor communication, inadequate teamwork, and deficiencies in education and training. Simulation-based education (SBE) has therefore emerged as an effective strategy to address these gaps and improve clinical outcomes [4–6]. The use of simulation training to gain clinical experience in PPH management may provide an opportunity to improve safety and minimize medical malpractice.

SBE is defined as an educational technique with a high level of reality using role-play scenarios or high-tech models that are very close to real-life clinical situations. [7]. It enables healthcare workers to practice without harming the person and is an important tool for team education to increase safety [8]. SBE helps to improve case response times [9, 10] and performance [11] and provides a safe environment for individuals to practice continuously and in a planned manner to improve their clinical skills [12, 13]. A study in the United States concluded that SBE given to experienced clinical teams significantly improved PPH intervention times [14].

PPH is one of the major maternal mortality and morbidity threats faced by healthcare professionals [9]. Midwives are the professional group most likely to encounter PPH in the emergency department, labor, and delivery or postnatal wards. Therefore, it is important that midwives are competent in the management of PPH and have practical skills to make appropriate decisions and implement emergency care [11]. SBE for obstetric emergencies is known to improve knowledge and performance [11]. The use of simulation for emergency obstetric care in midwifery education should contribute positively to the learning processes and professional practices of midwives and students [15]. The frequency of the use of simulation in the process of clinical practice and skill development in midwifery education is increasing [16]. In a study investigating the effectiveness of SBE for midwives on performance and knowledge for PPH management, the authors found a significant increase in performance and knowledge after SBE [11]. The need to practice in a safe environment is critical for the management of high-risk conditions such as PPH. Rapid assessment, intervention, teamwork, and a well-functioning system are required to improve outcomes. The aim of this study was to identify various experiences, such as physical, emotional, and environmental, of participants in managing difficult cases such as PPH with SBE. This study was designed to explore whether the skills of students in a master’s program who worked as midwives could be improved with SBE and whether reality shock could be reduced in cases of probable PPH.

### Aim

The aim of this study was to explore the experiences of midwives who received SBE in PPH management.

## Method

### Design

The study was qualitative in nature using a descriptive phenomenological approach [17] to determine the effect of SBE using PPH scenarios on midwives' experiences of PPH management. Phenomenological research makes it possible to theorize the meaning of people's experiences.

Descriptive phenomenology investigates how people experience and describe events and situations through their senses. It reveals a basic framework that describes the layers of relationships, thoughts, feelings, and behaviors as they are [18]. The basic assumption here is that we can only know what we experience. Since understandings derives from sensory experiences, we must describe, explain, and interpret these experiences [19, 20]. The question that guided this study is as follows: “How did SBE with PPH management scenarios affect midwives' feelings, attitudes, and thoughts?” The study was reported according to the SRQR checklist.

### Participants

To identify midwives who would participate in the education, a course announcement was posted on social media groups of midwives who were master's students. Course applications were received for about a month. Seventy master’s students applied to attend the course. The midwives were ranked according to their grade point average and the students who would participate in the education were selected. The 18 students selected as participants were midwives living in nine different provinces of Türkiye. In Türkiye, the master's program in midwifery is two-year postgraduate education. Master's students can also work as midwives in the clinic [21, 22]. Purposive sampling method was used. Participants who were both employed and enrolled in a master's program were selected to broaden their clinical experience and enhance their skills. Participants who could speak and understand Turkish, who had at least one year of clinical experience as a midwife, who had experience working in the birthing room or postnatal room, who had experience in assisting birth, who were enrolled in a master’s program and who had not previously received SBE in PPH were included in the study.

Prior to the course, a list of backup participants was prepared in case any selected midwives withdrew. All 18 primary participants were contacted before the training to confirm their participation, and no backup candidates were required. During the study, three midwives withdrew for personal reasons, and their data were excluded from the analysis. Data saturation was observed after interviews with ten participants, as no new themes or responses emerged. Data collection continued up to 15 participants to ensure completeness, at which point no additional responses were obtained, confirming that data saturation had been achieved. Therefore, the number of participants was considered sufficient, and the data collection phase was concluded [23, 24].

### PPH management with SBE and the content

PPH management with SBE was conducted by expert trainers for a total of 16 h in two days, i.e., 8 h per day. The education given to midwives included 8 h of theoretical training followed by 8 h of simulation practice. The SBE program was financially supported by The Scientific and Technological Research Council of Türkiye (TÜBİTAK). The trainers were certified academics who were experienced in SBE. Information about the PPH management with SBE was published via a website (URL: https://postpartumkanama.wixsite.com/website). Participants experienced five different PPH scenarios. A path was followed starting from easy cases to difficult/complex cases. Each scenario was performed in approximately 15–20 min and participants were allowed to rest and step out of the role for 5–10 min. After each scenario, debriefing sessions lasting approximately 15–20 min were held with experienced academics. Video recordings were taken during the implementation of the scenarios and they were shown to the participants during the debriefing phase. Thus, the participant midwives had the opportunity to monitor and evaluate themselves. The program was designed as a training and experiential learning opportunity; there were no formal pass/fail criteria. Participants who attended all theoretical and simulation sessions and engaged in debriefings were considered to have completed the program.

### Data collection

Data were collected between December 15 and 16, 2022, using a semi-structured interview with an in-depth bilateral interview technique following the SBE with PPH management course.

The interview schedule consisted of a personal information form and semi-structured open-ended questions. Midwives who participated in the PPH management with SBE at Selçuk University, Selçuk Simulation Center were included in the study. The 600-square-meter center provides training for health professionals and health sciences students. It includes a labor and delivery room, a neonatal unit, adult patient rooms, a basic skills laboratory, and areas for debriefing and technical training, as well as a classroom for theoretical lessons. Various simulators are available; for this course, the Lucina manikin from CAE Healthcare was used specifically for managing obstetric emergencies through simulation scenarios. The purpose of the study was explained to the midwives. They were informed that the interviews would be recorded and their written informed consent was obtained. As the interviews required privacy, one-to-one interviews with the midwives were recorded. Interviews were conducted in a suitable room of the center (quiet and spacious) for approximately 30–35 min. During the data collection, two researchers interviewed one midwife. One researcher conducted the interview and one researcher acted as a reporter and audio recorded. The reporter recorded findings specific to the participants' expressions such as voice and facial expressions.

### Data collection forms

#### Personal Information Form

An 11-question self-report questionnaire form including descriptive characteristics of midwives, such as age, marital status, income level, unit of employment, years of employment, was prepared by the researchers by reviewing the literature [4, 25, 26].

#### Semi-structured interview form

It consisted of 10 open-ended questions that explored the experiences of midwives after PPH management with SBE. The form was developed by the researchers based on the literature [10, 11, 25, 26]. Examples of the interview questions were as follows: *"How did you feel during the simulation exercise?, Can you tell us about your experiences with this SBE?, What was the event/element that affected you the most during the SBE?, When you think about what you heard, saw, or touched during the SBE, what were the things that affected you the most?, How did the behavior of other people in the scenario during the SBE affect your perception of the situation?".*

#### PPH management performance evaluation form

It consists of 15 items questioning the performance evaluations of midwives after PPH management with SBE by reviewing the literature by the researchers [2, 3, 11]. The items were filled in by the researchers during simulation-based scenarios of PPH management practices. The midwives' behaviors regarding each item were taken into consideration and scored as “0 (Did not apply), 1 (Moderately applied), or 2 (Well applied)”. This evaluation form was used for student observation purposes only. The scores on the form were not tallied or used as passing scores. Participants were not required to repeat scenarios if they did not receive a perfect score. However, each participant had the opportunity to observe all scenarios in which they and other midwives participated. This provided additional observational learning and skill reinforcement. The study's design intentionally avoided pass/fail labels to prevent stress and discomfort for participants.

### Statistical analysis

Descriptive statistics were evaluated using the Statistical Package for Social Science 20 (SPSS 20.0) program for the Personal Information Form and the PPH Management Performance Evaluation Form. Thematic analysis was carried out based on the descriptive phenomenological approach. Meaning was extracted from the original data, organized into patterns, and the results of the themes were written up in relation to the purpose of the study and the real context [17]. Two researchers in the study transcribed the information in the interview forms and audio recordings on an electronic text file. The other two researchers checked the reports and transcripts of the audio recordings. Each researcher then independently read and analyzed all interviews. The interview transcripts were read in detail to capture and define the meanings (categories) emerging from the data. In the second reading, the researchers gradually identified the relationships between categories and subcategories based on context. Individual analyses were then examined comparatively. Differences were discussed in detail and a consensus was reached. The categories identified by the two researchers were listed and then reviewed by two other researchers, thus increasing the degree of inter-rater reliability. Finally, the findings were organized into themes that included the researchers' notes and excerpts from the participants' narratives. Thus, the meanings obtained from the participants' experiences were explained in meaningful terms and organized into themes. Main and sub-themes were formed by determining the consistency between the identified themes. The themes were sent to five academics who are experts in the field of midwifery for their opinions. The final version of the themes was created in line with the opinions. Midwives' experiences with SBE for PPH management were explored as the four main themes: “simulation experience, awareness formation, emotional effects, and effects of team members”. The document portrait and code map of the themes explored as a result of the qualitative data analysis were created using the MAXQDA 11.0.2. (VERBI Software, Berlin 2014 (v1.2)) program [27]. The abbreviation "Midwife (M)" was used for the opinions of the midwives who participated in the study.

### Ethical statements

Ethical approval was obtained from the Non-Interventional Clinical Research Ethics Committee of a university (Date: December, 2022, Decision No: 2022/1245). Participants were not required to give names while filling out the surveys. An explanation about the study and an informed consent form was provided on the first page of the questionnaire and approved by the participants.

## Results

Among the midwives who participated in the study, 66.7% worked in secondary health care institutions, while 33.3% were assigned to delivery rooms. Of the midwives, 73.3% were working day and night shifts for an average of 176.0 ± 23.56 h per month. Fifty-four percent of the midwives encountered a PPH case and 66.7% had knowledge about SBE (Table 1).

**Table 1:** Descriptive characteristics

Descriptive Characteristics		n	%
Age	Mean ± SD = 27.0 ± 7.92, Min = 24, Max = 49 years		
Marital Status	Married	6	40.0
	Single	9	60.0
Monthly Income Status	Income less than expense	2	13.3
	Income equals to expense	10	66.7
	Expense more than income	3	20.0
Organization	Primary health care institutions	1	6.7
	Secondary health care institutions	10	66.7
	Tertiary health care institutions	4	26.7
Hospital Unit	Emergency	2	13.3
	Perinatology	1	6.7
	Delivery Room	5	33.3
	Postpartum Service	1	6.7
	Other (District Health Directorate Mental Health Unit, Intensive care, NST polyclinic, Family health center)	6	40.0
Shift	Only daytime	4	26.7
	Day and Night	11	73.3
Monthly Working Hours	Mean ± SD: 176.0 ± 23.56, Min: 120, Max: 230 h		
Experience of encountering a PPH case	Yes	8	53.3
	No	7	46.7
Frequency of encountering a PPH case	Rarely	4	50
	Sometimes	4	50
The state of knowledge about simulation	Yes	10	66.7
	No	5	33.3
Total		15	100

*SD* Standard Deviation, *Min* Minimum, *Max* Maximum, *NST* Non-Stress Test, *PPH* Postpartum Hemorrhage

During simulation-based scenarios of postpartum hemorrhage management practices, 9 (60%) of the midwives were able to detect postpartum hemorrhage, 15 (100%) were able to assess the amount of bleeding, and 9 (60%) performed uterine evaluation. Furthermore, 4 (26.7%) of the midwives did not check and empty the bladder when necessary, and 10 (66.7%) did not check for tears in the perineum, cervix, and vagina (Table 2).

**Table 2:** Item-based performance scores of midwives during the simulation-based scenarios of postpartum hemorrhage management practices

Item	Point	n	%
Detecting the condition for postpartum hemorrhage	2	9	60.0
	1	4	26.7
	0	2	13.3
Providing leadership and delegation of tasks	2	5	33.3
	1	10	66.7
	0	0	0
Trying to provide additional assistance	2	8	53.3
	1	5	33.3
	0	2	13.3
Taking the mother's history	2	7	46.7
	1	8	53.3
	0	0	0
Informing the mother about the procedures to be performed	2	11	13.3
	1	2	13.3
	0	2	73.3
Keeping the mother calm and allowing her to express her fears and concerns	2	12	80.0
	1	1	6.7
	0	2	13.3
Measure and observe vital signs (BP, pulse, temperature and respiratory rate) to understand mother's physical condition	2	11	73.3
	1	4	26.7
	0	0	0
Assessing the amount of bleeding	2	15	100
	1	0	0
	0	0	0
Supine positioning to reduce blood flow to the uterus	2	9	60.0
	1	6	40.0
	0	0	0
Performing first interventions to stabilize the condition (intravenous line is opened, 4–6 O_2_ liters/minute cardiac monitoring)	2	5	33.3
	1	6	40.0
	0	4	26.7
Selecting and administering an appropriate infusion	2	15	100
	1	0	0
	0	0	0
Checking the bladder and emptying it if necessary	2	9	60.0
	1	2	13.3
	0	4	26.7
Evaluating the uterus (massaging the loose fundus by palpation to make it contract)	2	9	60.0
	1	6	40.0
	0	0	0
Checking for tears in the perineum, cervix, and vagina	2	2	13.3
	1	3	20.0
	0	10	66.7
Administer oxygenation using an appropriate dose and method	2	5	33.3
	1	6	40.0
	0	4	26.7

**Fig. 1:**
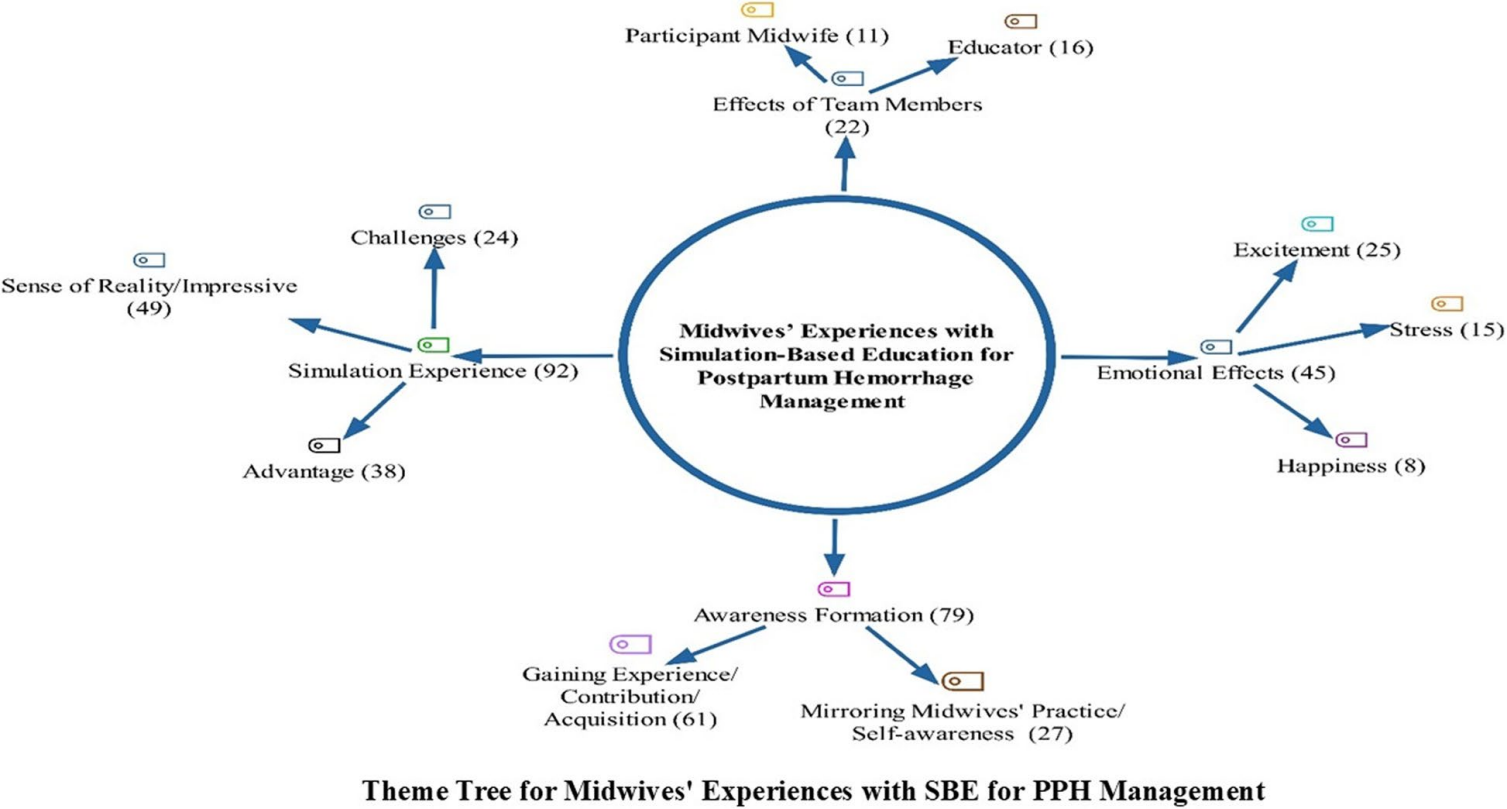
Theme tree for midwives’ experiences with SBE for PPH management

The researchers examined the responses of the midwives in depth and made a total of 520 codes. Four main and ten sub-themes were formed by combining the codes. Midwives' experiences with SBE for PPH management were determined as the main themes of “simulation experience, awareness formation, emotional effects, and effects of team members”. The main theme and sub-themes are shown as a theme tree in Fig. 1. When the coding numbers were examined, the main themes of “simulation experience” with 92 coding numbers and “awareness formation” with 79 coding numbers were the most emphasized themes (Fig. 1).

### “Simulation experience” Theme

The main theme of midwives' SBE experience consists of the sub-themes of “sense of reality/impressive, advantage and challenges”. The most emphasized sub-theme of the SBE experience main theme was “sense of reality/impressive” with 49 codes. Midwives stated that they were affected by the model's speech, changes in vital signs, bleeding, and the baby’s crying during the simulation. They stated that this SBE gave them experience in intervening in real-life cases. For example, midwives said *(M2) “During the simulation, I was impressed by the similarity of the baby to a real baby… it was exciting to see the simulator bleeding, vital signs changing after drug administration and the woman giving feedback.”, (M11) “I was very impressed by the simulator's movements, eye movements, speech, feeling of the pulse and the real-life-like vibe of the limbs. Its reactions to our applications gives the feeling of being in a real event.”, and (M10) “I was excited and thought it was real and felt my hands and feet shaking. The fact that the woman was bleeding a lot was the situation that affected me the most and I thought I had to quickly plan what to do. During the practice, I felt like I was working, not in a simulation. I can't even describe my emotions… it was one of the most enjoyable moments in my life.”*

Most of the midwives stated that the PPH management with simulation-based educational scenarios looked so real that they could encounter in the clinic*.* For example, midwives mentioned *(M7) “It was very good that the model responded to the physical applications and the simulation scenario was realistic enough to be encountered in the clinic. I felt the atmosphere of a room with a real woman with PPH to the fullest.”, (M9) “I felt that I was experiencing exactly the same situation as I was in the clinic during the simulation. The bleeding of the woman was exactly the same as the reality. It was impossible not to be impressed.”*

### “Awareness formation” Theme

In this study, the main theme of awareness formation of midwives consists of the sub-themes of “gaining experience/contribution/acquisition and mirroring midwives’ practice/self-awareness". Midwives evaluated themselves after the SBE and realized their correct or incorrect practices. By making self-evaluations they realized that they had deficiencies in the following aspects: monitoring vital signs more frequently, calling for help, taking the woman's relatives out of the room, recording the practices, team coordination, and communication during the practice. For example, midwives stated *(M5) “Being able to see the deficiencies and differences with practical application affected me positively. I could have taken the woman's anamnesis in more detail. I should have monitored vital signs more frequently.”, (M6) “I could have done the vaginal examination better during the practice and acted more calmly. I could have practiced step by step according to the symptoms of postpartum bleeding.” (M12) “I think I need to improve myself in controlled cord traction and uterine massage practices and to work more actively and consciously in teamwork.” and (M14) “First of all, I understood the importance of woman communication and informing the physician in emergencies. I realized my deficiencies in crisis management and team coordination.”*

### “Emotional effects” Theme

The main theme of emotional effects of midwives consists of “excitement, stress, and happiness” sub-themes. Most of the midwives stated that they felt the feeling of excitement very much because they practiced with simulation for the first time. While some of the midwives stated that they suppressed the feeling of excitement and adapted to the practices, others stated that they were stressed and felt incomplete in the practices. For example, midwives said *(M1) “I intervened with a sense of reality. The fact that there were relatives inside stressed me out. We couldn't even see the changes in vital signs because of the excitement. I tried to suppress my excitement, in fact, if we weren't so nervous, we could do the interventions better.”, (M4) “I was excited that the woman did not stop bleeding despite interventions in case management. While I was thinking about what medication to give, the woman’s condition got worsened. I felt helpless.”, (M5) “Of course, I experienced a great excitement. A slight fear of the unknown followed, but as soon as I started working on the case, I calmed down a bit as I felt like there was a real case.”, and (M10) “I was shocked at first when I saw the bleeding, but I quickly got rid of this feeling and started thinking about what I should do. I was scared when I saw that the woman had no vital signs. I was afraid of losing the woman.”*

The midwives felt happy to experience SBE and that they would not harm the woman even if they made a mistake. They also think that their practice skills have improved because they have the chance to do the same practice again. For example, one midwife said *(M9) “Practicing on the simulation made me feel comfortable because it was not a real woman and I would not harm even if I made a mistake.”*

### “Effects of team members” Theme

In this study, the main theme of the effects of team members consists of the sub-themes of "participant midwife and educator". During the scenario implementation, the communication and harmony of the two midwives with each other and the guidance of the educator according to the situation by taking a role in the scenarios affected the practices. Some midwives were uncomfortable with the presence of the educator in the simulation, panicked and felt more excitement. For example, some midwives mentioned *(M8) “Our communication with my midwife friend I worked with progressed very well. During the SBE, I thought about what I should do according to the application steps and felt that I should be in teamwork during the intervention.”, (M9) “Teamwork is always very important in emergencies. People in the team need to be calm. I think task sharing and calmness, minimizing stress and one person should lead and distribute tasks to other health professionals. I could have taken the woman's relatives outside, I forgot in panic.”, and (M11) “The reactions of the mother and the reactions of her partner around her affect what to do at that moment. While the reactions of the mother help me to focus on her, the involvement of her husband can have a negative effect. For this reason, I think that in case of bleeding, the spouse should be removed from the room.”*

Some midwives stated that the practice was like in real life with the presence of the trainer in the simulation scenario as the women's relative. For example, some midwives stated (M12) *“The behaviors of the people in the scenario about midwifery practices were very informative about how I should manage the situation. It gave me an idea about what should be done and what should not be done. Having a woman relative in the room made it more realistic.”, and (M13) “I think the presence of other people in the scenario allows us to perceive the practice more realistically.”*

## Discussion

The present study examined the experiences of midwives with SBE in the management of PPH. The study observed that midwives successfully implemented most of the procedural steps during performance assessments. In performance evaluations such as informing the mother about the procedures to be performed, keeping the mother calm, enabling her to express her fears and concerns, assessing the amount of bleeding, placing the woman in the supine position to reduce blood flow to the uterus, selecting and administering the appropriate infusion, it is seen that most of the midwives performed these practices. Similarly, Kato and Kataoka (2017), in their SBE study of PPH management with midwives, found that midwives' performance and knowledge levels increased significantly after SBE [11]. In other studies of SBE for PPH management, implementation skills scores increased in groups receiving SBE [4, 28–31].

Four main themes (simulation experience, awareness formation, emotional effects, and effects of team members) developed in the study regarding midwives' simulation-based educational experiences in PPH management. In a similar study, educational aspects of SBE, perceived competence, and motivational and emotional aspects [9] were the main themes.

In this study, the main theme of “simulation experience” consists of sub-themes of sense of reality/impressive, advantage and challenges. The sense of reality/impressive sub-theme was the most emphasized theme. Summer et al. (2022) investigated SBE for the management of PPH and reported that SBE was educational, relevant, and realistic; the debriefing session provided an important learning opportunity; and it was a safe learning environment [32]. In this study, midwives stated that the features of the models used in SBE, such as giving realistic reactions, having vital signs, and the pregnant woman bleeding, gave the feeling that they were practicing PPH management in a real clinical setting. The midwives also stated that the scenarios they played were like real scenarios that they were likely to encounter in the clinic. Studies in the literature also show that simulation education is more engaging, interactive, fun [28, 33], comfortable [10, 34], and associated with increased satisfaction [35].

The main theme of midwives' “awareness formation” in this study consists of the sub-themes of gaining experience/contribution/acquisition and mirroring midwives’ practice/self-awareness. Midwives had the opportunity to evaluate themselves and realize their mistakes after SBE. A study reported that SBE creates a higher sense of self-efficacy in individuals because it increases knowledge and confidence in PPH management. Perceived self-efficacy increases significantly with SBE in obstetric emergencies, leading to improved performance [9]. In this study, midwives stated that they realized their deficiencies in the practices to be performed during the scenarios, increased their skills in coping with stress and acting in coordination with their teammates, and that they would perform the PPH management steps more carefully when faced with a PPH situation in the future. Dillon et al. (2021) conducted a study to evaluate whether the implementation of a SBE program for PPH improved clinical performance and outcomes. They found that there was less blood loss in PPH after the SBE and that this was associated with improved woman outcomes [36].

On the other hand, a study on the subject reports that the experience of scenarios in SBE significantly reduces stress levels and increases confidence [9]. Another study shows that SBE increases students' confidence, knowledge, and skills [37]. In this study, midwives stated that practicing on simulation made them feel comfortable, happy, and excited because there was no harm to the real women. They also stated that although they felt stressed at the beginning of the scenarios, their stress levels decreased in the following minutes and their adaptation increased. Alsaraireh et al. (2024) reported in their study that SBE significantly increased students' knowledge, satisfaction, and confidence and that this can be a successful teaching approach to improve students' knowledge, satisfaction, and confidence [38]. In this study, the main theme of “emotional effects” consists of excitement, stress, and happiness sub-themes. While some of the midwives stated that they suppressed their excitement and adapted to the practices, others stated that they were stressed and had deficiencies in the practices. In the study, midwives felt happy that they would not harm the woman if they made mistakes during SBE. Their skills improved because they had the chance to repeat the applications. In PPH, which is a life-threatening complication, it is important to know how to perform interventions and to perform the procedure on time. Repeated applications ensure that the correct intervention can be applied on time, which builds confidence in the person [9]. Repetitive practice is considered one of the most important factors for improved self-efficacy [39]. In addition, studies have shown that repeated interventions increase midwifery students' confidence [40] and support them [41]. Therefore, SBE could help to better understand the practices and improve students' skills in general.

Finally, the main theme of the “effects of team members” consists of “participant midwife and educator” sub-themes. During the scenario implementation, the communication and harmony of the two midwives with each other affected their performance. In this study, midwives stated that teamwork is important in emergencies and that a safe environment can be provided, calmness can be maintained, and stress can be reduced by the distribution of tasks by the team leader. In addition, the fact that educators took part in the scenarios as woman relatives contributed to the fact that it was considered as a practice environment closer to reality by the participating midwives. The emphasis on the importance of team training is supported by previous research. Egenberg et al. (2017), in their qualitative study on PPH in healthcare personnel, showed that SBE enabled them to use a teamwork approach, collectively utilize their competencies and improve their response time to challenging situations. The opportunity for participants to discuss their roles and perceived responsibilities in a safe environment enabled them to improve their own practice and how to collaborate in cases of PPH [9].

### Limitations of the study

It is thought that the study may contribute to the literature as it is the first qualitative study investigating midwives' experiences of PPH management with SBE in Türkiye. However, the study was conducted in a single simulation center, which may limit the transferability of the findings to other settings. Due to the one-to-one application of SBE with a small number of groups, the research data are limited to 15 participants. Therefore, the results of the study cannot be generalized to all midwives. Another limitation is that each simulation scenario was performed only once. Repetition of the scenarios could have provided more comprehensive data to evaluate the development of midwives’ practical skills and learning processes. Future studies are recommended to design longer training programs with repeated scenarios, larger samples, and multi-center participation to enhance generalizability.

## Conclusion

As a result of the study, the main themes of “simulation experience, awareness formation, emotional effects, and effects of team members” were explored with regard to midwives' experiences with SBE for PPH management. The management of PPH with SBE has increased midwives’ awareness and experience, allowed them to undergo varied emotional responses, including stress and satisfaction, and improved their teamwork skills. In the PPH management course with SBE, midwives experienced a realistic simulated clinical environment by taking on roles in scenarios and gained application and communication skills by observing women’s reactions during midwifery care. They also learned how to coordinate teamwork in cases of PPH requiring urgent intervention. Feedback from the midwives following the SBE was mostly positive.

### Implication for practice

SBE is an effective and beneficial method of midwifery education. SBE contributes to the development of midwives' self-awareness, increases their self-confidence and satisfaction, allows them to practice in a realistic environment, and improves their skills. Performing and repeating practices without the risk of harming the individual allows them to gain experience. Midwives are the professional group most likely to encounter PPH cases. To improve midwives' practice skills and minimize malpractice, in-service SBE with scenarios should be implemented frequently.

## Data Availability

The research data is available upon request. To request the data, contact the corresponding author of the article.

## Abbreviations

M: Midwife
TÜBİTAK: The Scientific and Technological Research Council of Türkiye
PPH: Postpartum hemorrhage
SBE: Simulation-based education
